# Antibacterial Effects of Natural Herbal Extracts on* Streptococcus mutans*: Can They Be Potential Additives in Dentifrices?

**DOI:** 10.1155/2017/4921614

**Published:** 2017-10-19

**Authors:** Spoorthi Banavar Ravi, Sudarshini Nirupad, Prashanthi Chippagiri, Rohit Pandurangappa

**Affiliations:** ^1^School of Dentistry, International Medical University, No. 126, Jalan 19/155B, Bukit Jalil, Kuala Lumpur, Malaysia; ^2^Oral Pathology, Dr. Syamala Reddy Dental College Hospital and Research Centre, SGR College Main Road, Marathahalli Post, Bangalore, Karnataka, India; ^3^Faculty of Dentistry, MAHSA University, Kuala Lumpur, Malaysia

## Abstract

**Background:**

Many plants or herbs exhibit potent antimicrobial activity against various microorganisms. They have no side effects and presumably act against and modulate the factors that are crucial for microbial survival or their activity.* Streptococcus mutans* is a pioneer bacteria implicated in dental caries. This study aims to evaluate the antimicrobial activity of garlic bulbs, pudina leaves, and mango and eucalyptus twig extracts on* Streptococcus mutans* by evaluating their zone of inhibition and determining their minimum inhibitory concentration (MIC).

**Methods:**

Microbiological assay (well diffusion method) to determine zone of inhibition against pure forms of* Streptococcus mutans* was performed. The antibacterial effects of methanolic extracts of mango twigs, eucalyptus twigs, pudina leaves, and garlic bulbs were studied. Test compounds were further evaluated for their MIC.

**Results:**

Extracts derived from mango and eucalyptus twigs showed significant antibacterial effects at test concentrations. Pudina and garlic extracts did not show any significant antibacterial effects at similar concentrations. Upon further evaluation of the 2 positive compounds for their MIC, mango twigs demonstrated more antimicrobial potential than eucalyptus twigs at a lower concentration.

**Conclusion:**

Our observations indicated that the mango twig extracts possess higher antibacterial effects against* Streptococcus mutans* than other compounds at specific test concentration.

## 1. Introduction

Dental caries is a chronic microbial disease affecting humans in all parts of the world [1, 2].* mutans* group of streptococci, being the active cause of caries [3, 4], produce weak organic acids after fermenting carbohydrates, or a byproduct resulting in demineralization of the tooth structure [5, 6]. Preventing and controlling dental caries has been a great challenge. For ages many prophylactic agents have been used to prevent dental caries such as antibiotics, plant and herb derived compounds, mouth washes, tooth pastes, gels, varnishes, and the caries vaccines [7]. One such method practiced from an age-old time is use of natural herbs especially by rural people to clean their teeth. In our study, we aimed at evaluating the antimicrobial potential of natural herbs like garlic (*Allium sativum*), pudina (*Mentha arvensis*), mango (*Mangifera indica*) twigs, and* Eucalyptus* (*globulus* Labill., nilgiri) twigs on* S. mutans*.

## 2. Materials and Methods

The materials procured for this in vitro test compounds were garlic lobes, pudina leaves, mango twigs, and eucalyptus twigs. The test bacteria were* Streptococcus mutans* ATCC 2517 (American-type culture collection). Mueller Hinton agar plate and Tecan plate reader were used.

### 2.1. Preparation of Alcoholic Extract of Test Compound

Preparation of alcoholic extract of each test compound was done using 10 g of dried and powdered material added to 50 ml methanol and incubated at 50°C for 4 hours and then filtered through Whatman filter paper. The supernatant was dried at 80°C. A thick paste was obtained that yielded approximately 5–7% of extracts; that is, for 10 g of test compound it yielded 0.5–0.7 g or 500–700 mg of extract. The stock preparation was done using 100 mg/ml in methanol. Standardization was based on the NCCLS (National Committee for Clinical Laboratory Standards) method. The microorganisms,* Streptococcus mutans* (ATCC 2517), were obtained from American-type culture collection, USA. The inoculum was cell suspension prepared from cultures grown on trypticase soy broth adjusted to 1.2 × 10^5^. The positive control was chlorhexidine mouth wash-0.2% [8–11] and ciprofloxacin 250 mg [12] and the negative control was methanol.

The test was carried out in two steps, that is, evaluation of antimicrobial activity by evaluating zone of inhibition by well diffusion method and determination of minimum inhibitory concentration (MIC) as per NCCLS method [13, 14].

#### 2.1.1. Evaluation of Antimicrobial Activity by Zone of Inhibition by Well Diffusion Method

100 *μ*l inoculum of test bacterial cultures was inoculated on Mueller Hinton agar plates (90 mm). The test compounds (1 mg and 2 mg, 10–20 *μ*L) and test drugs chlorhexidine (40 *μ*g, 20 *μ*L) and ciprofloxacin (2.5 *μ*g, 20 *μ*L) were impregnated on 5 mm wells. The plates were incubated at 35°C for 24–48 hours and observed for zone of inhibition around the well.

#### 2.1.2. Determination of MIC (Minimum Inhibitory Concentration) as per NCCLS Method

The test bacteria, that is,* Streptococcus mutans* (ATCC 25175), and the test compounds, that is, methanolic extract of eucalyptus twigs and mango twigs, were used at the concentration of 16–1024 *μ*g/ml by twofold dilution method in Mueller Hinton broth. Test drugs were Ciprofloxacin and Chlorhexidine used at the concentrations of 0.25–16 *μ*g/ml and 4–256 *μ*g/ml, respectively, by twofold dilution method in Mueller Hinton agar broth. The test compounds and the test drugs (positive control) were diluted for 8 different concentrations by 2-fold dilution method [8, 9]. Ninety ml drug or test compounds of different test concentration was mixed with 10 ml inoculum in 96-well plates in triplicate. The control used was 90 ml Mueller Hinton agar without drug mixed with 10 ml inoculum, all incubated at 35°C. The bacterial test plates were observed after 24–48 hours. Optical density (OD) of 600 nm was measured in a Tecan plate reader. MIC (minimum inhibitory concentration) was determined at 50% inhibition of OD as compared with control.

## 3. Results

**Figure 1:**
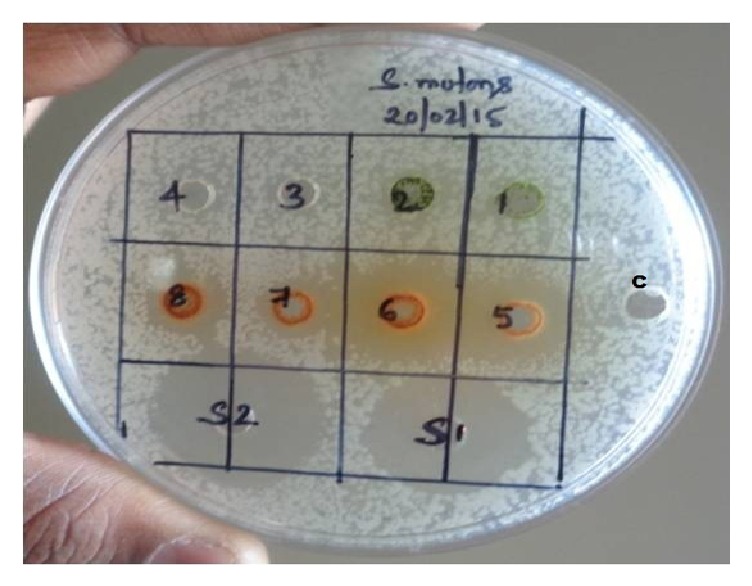
Inhibitory activity of test samples on* Streptococcus mutans*. Test extracts: (1) pudina (1 mg/well), (2) pudina (2 mg/well), (3) garlic (1 mg/well), (4) garlic (2 mg/well), (5) mango (1 mg/well), (6) mango (2 mg/well), (7) eucalyptus (1 mg/well), (8) eucalyptus (2 mg/well), S1: chlorhexidine (40 *μ*g/well), S2: ciprofloxacin (2.5 *μ*g/well), and C: methanol control (negative).

**Figure 2:**
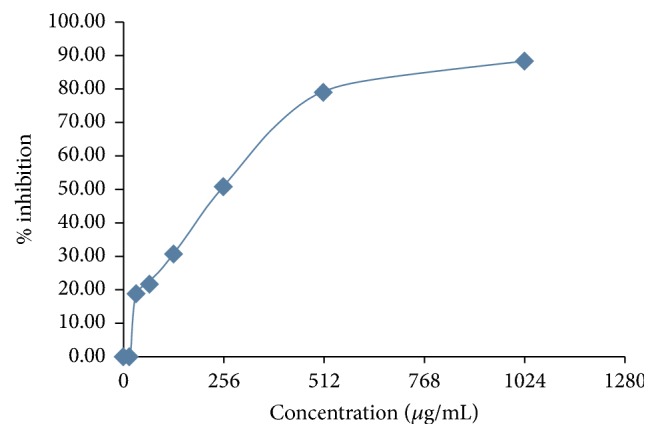
Inhibitory activity of mango twigs extracts against* S. mutans*.

**Figure 3:**
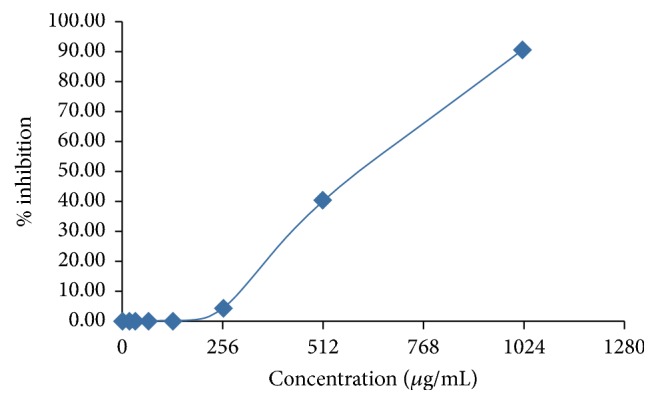
Inhibitory activity of eucalyptus twigs extracts against* S. mutans*.

The zone of inhibition was observed against positive controls and test sample after incubating at 35°C for 24–48 hours (Figure 1). The mango and eucalyptus twig extracts clearly showed larger zone of inhibition in comparison with other test compounds at particular concentration, as indicated/summarized in Figure 1 and Table 1. Mango twig and eucalyptus twig extracts were further evaluated to know their minimum inhibitory concentration (MIC). This was done along with determination of minimum inhibitory concentration of standards (ciprofloxacin and chlorhexidine) against* Streptococcus mutans*. The MIC observed for ciprofloxacin was at concentration of 0.5 *μ*g/ml and the percentage of inhibition was 79.28 and MIC for chlorhexidine was at concentration of 4.00 *μ*g/ml and the percentage of inhibition noted was 87.50 (Table 2). The MIC recorded for mango twig extract was at concentration 256 *μ*g/ml and the percentage of inhibition was 50.70 and MIC for eucalyptus extract was at concentration 1024 *μ*g/ml and the percentage of inhibition was 90.90 (Table 3, Figures 2 and 3).

## 4. Discussion

Dental caries is an irreversible chronic disease initiated by* Streptococcus mutans*, a Gram-positive, facultative anaerobic microorganism [15]. Preventing and controlling dental caries have been a great challenge for decades. The garlic extract (*Allium sativum*) can inhibit growth of both Gram-positive and Gram-negative bacteria. The garlic cloves consist of sulfur containing chemicals like allicin, alliin, and ajoene [16]. When the garlic cloves are cut or crushed they release the enzyme alliinase which converts alliin to allicin and allicin is responsible for antibacterial activity [8]. The anticariogenicity of garlic extract was evaluated in previous studies and can inhibit the bacterial growth only when used at higher concentration [8, 9, 15, 17]. Pudina or mint leaves are widely used in food and medicines for their flavor and antibacterial effect. Mint leaves are comprised of menthol, menthone, methyl esters, and terpenoids which are responsible for antibacterial effect [18]. Menthol, being the chief component [19], is used in essential oils and ointments [20]. The antibacterial activity of alcoholic extract of pudina leaves was previously evaluated by Chaudhary et al. at three different concentrations, 5%, 10%, and 50%. The zone of inhibition was seen at 10% and 50% concentrations [10]. In another study, the alcoholic extract of mint leaves at 10 mg/ml was more active against microorganism [12]. Antibacterial properties of alkaloid extracts from* Callistemon citrinus* and* Vernonia adoensis* against* Staphylococcus aureus* and* Pseudomonas aeruginosa* were evaluated by Mabhiza et al. in 2016 and they reported that alkaloids may serve as potential courses of compounds that can act as lead compounds for the development of plant-based antibacterials and/or their adjunct compounds [21]. In our study, to evaluate the antibacterial effect on* Streptococcus mutans* we used garlic lobes (*Allium sativum*) and pudina leaves (*Mentha arvensis*) alcoholic extract at 1 mg/ml and 2 mg/ml concentrations; both the test compounds did not show any antibacterial effect against the cariogenic bacteria. The reason for this could be the low concentration of test extracts that were used (1 to 2 mg/ml).

 Mohammed used eucalyptus leaves and revealed that methanolic extract of* Eucalyptus spathulata *leaves was more effective in inhibiting* Streptococcus mutans* compared to gentamycin and nystatin [22].* Eucalyptus spathulata* twig consists of ketones like juglone, regiolone, sterol, and flavonoid comprising antibacterial potential [23]. We used methanolic extract of eucalyptus twigs that demonstrated antimicrobial activity against* Streptococcus mutans* compared to garlic lobes extract and pudina leaves extract.

The* Mangifera indica* consists of tannins, bitter gum, and resins [24]. The tannins and resins have astringent effect on mucous membrane; they protect enamel by forming layer on it. Prashant et al. used mango twigs as one of the test compounds to test the antimicrobial activity of* Streptococcus mutans*,* Streptococcus salivarius*,* Streptococcus mitis*, and* Streptococcus sanguis*. At 50% concentration it showed maximum zone of inhibition on* Streptococcus mitis* [25]. In our study when we used mango (*Mangifera indica*) and* Eucalyptus* (*globulus* Labill.) twig extract, at concentrations of 1 mg/ml and 2 mg/ml, the mango twig extract showed highest zone of inhibition compared to extract of eucalyptus twig. When further evaluated for minimum inhibitory concentration, mango twig extract showed inhibitory activity at minimum concentration of 256 *μ*g/ml compared to eucalyptus twig methanolic extract which was 1024 *μ*g/ml.

The tested compounds* Mangifera indica* and eucalyptus that showed highest antibacterial activity at minimum concentration can be incorporated in oral rinses, dentifrices, cavity liners, and varnishes to improve oral hygiene and cleanliness. These are easily available and are economical.

The effectiveness of chitosan shell toothpaste white shrimp (*Litopenaeus vannamei*) in reducing* Streptococcus mutans* in cases of early childhood caries was evaluated by Achmad and Ramadhany [26], by counting the number of colonies formed before and after using the toothpaste. Their results indicated a significant reduction of the number of colonies of* Streptococcus mutans* in the case of early childhood caries. Widyagarini et al. [27] in 2016 tried to identify serotypes c and e* Streptococcus mutans* in child-mother pairs and determine the relationship between serotype of* S. mutans* and dental caries in plaque samples. There was no significant relationship between serotype c/e* S. mutans* and child-mother caries score.

The limitations of our study include that this study was conducted in vitro with the extracts of mango and eucalyptus twigs. The duration of the contact of such extracts with the microorganisms in the oral cavity in vivo is not clear; hence further studies comparing the prevalence of dental caries among users and nonusers of such extracts from the twigs should help elucidate the picture. The tested compounds can be further tested to know their minimal bactericidal concentrations (MBC) to comprehensively understand their efficacy against the most dreaded bacteria causing the dental caries.

## 5. Conclusion

The results of our study indicate that the mango twigs possess the antibacterial effect even at low concentration against the most cariogenic bacteria* Streptococcus mutans*. It appears that it may be possible to combat* Streptococcus mutans* to increase the efficacy of the oral hygiene practices by incorporating the mango and eucalyptus twig extracts into dentifrices. However, studies simulating in vivo situations more closely are required to get a clear understanding.

## Figures and Tables

**Table 1:** Inhibitory activity of Test samples on Streptococcus mutans.

Test compound	Concentration (mg/Well)	Zone of inhibition (in mm) Test organisms *Streptococcus mutans*
Pudina	1	—
	2	—
Garlic	1	—
	2	—
Mango extract	1	13.5 ± 0.5
	2	16.0 ± 1.0
Eucalyptus extract	1	9.5 ± 0.5
	2	11.25 ± 0.25
Chlorhexidine	0.040	20.0 ± 0.0
Standard ciprofloxacin	0.0025	23.5 ± 0.5

**Table 2:** Minimum inhibitory concentration of ciprofloxacin and chlorhexidine against *Streptococcus mutans*.

Ciprofloxacin concentration (*µ*g/ml)	% *inhibition*	Chlorhexidine concentration (*µ*g/ml)	% *inhibition*
0.00	0.00	0.00	0.00
0.25	48.91	4.00	87.50
0.5	79.28	8.00	88.27
1.0	81.00	16.00	91.24
2.0	88.30	32.00	94.79
4.0	88.05	64.00	95.05
8.0	94.49	128.00	96.64
16.00	92.65	256.00	97.71
0.00	81.00	0.00	88.27
MIC	0.5	MIC	<4

**Table 3:** Minimum inhibitory concentration of mango and eucalyptus twigs extract against *Streptococcus mutans*.

Concentrations (*µ*g/ml)	% inhibition	
	Mango twigs extract	Eucalyptus twig extracts
00	00	0.00
16	0.00	0.00
32	18.52	0.00
64	21.71	0.00
128	30.57	0.00
256	50.70	4.35
512	79.23	40.55
1024	88.62	90.90
MIC	256	1024
